# Short-Chain Chitin Oligomers: Promoters of Plant Growth

**DOI:** 10.3390/md15020040

**Published:** 2017-02-15

**Authors:** Alexander J. Winkler, Jose Alfonso Dominguez-Nuñez, Inmaculada Aranaz, César Poza-Carrión, Katrina Ramonell, Shauna Somerville, Marta Berrocal-Lobo

**Affiliations:** 1 Department of Systems and Natural Resources, MONTES (School of Forest Engineering and Natural Environment), Universidad Politécnica de Madrid, Ciudad Universitaria s/n, 28040 Madrid, Spain; alexander.winkler@mpimet.mpg.de (A.J.W.); josealfonso.dominguez@upm.es (J.A.D.-N.); 2 Department for Wood Biology, Centre for Wood Science and Technology, Universität Hamburg, Leuschnerstr. 91d, D-2103 Hamburg, Germany; 3 Departamento de Físico-Química, Instituto de Estudios Bifuncionales, Facultad de Farmacia, Universidad Complutense, Paseo Juan XXIII, 1, 28040 Madrid, Spain; iaranaz@hotmail.com; 4 Centro Nacional de Biotecnología, Calle Darwin, 3, 28049 Madrid, Spain; cpoza@cnb.csic.es; 5 Department of Biological Sciences, P.O. Box 870344, University of Alabama, Tuscaloosa, AL 35487, USA; kramonel@bama.ua.edu; 6 Plant Biology, Carnegie Institution of Science, 260 Panama St., Stanford, CA 94305, USA; ssomerville@berkeley.edu; 7 Centro de Biotecnología y Genómica de Plantas, Instituto Nacional de Investigación y Tecnología Agraria y Alimentaria (INIA), Campus Montegancedo UPM, Universidad Politécnica de Madrid (UPM), 28223 Pozuelo de Alarcón (Madrid), Spain

**Keywords:** chitin oligosaccharides, bio-stimulator, fertilizer, soil biomass, biodiversity, soil health, soil biomass, bio-diversity

## Abstract

Chitin is the second most abundant biopolymer in nature after cellulose, and it forms an integral part of insect exoskeletons, crustacean shells, krill and the cell walls of fungal spores, where it is present as a high-molecular-weight molecule. In this study, we showed that a chitin oligosaccharide of lower molecular weight (tetramer) induced genes in *Arabidopsis* that are principally related to vegetative growth, development and carbon and nitrogen metabolism. Based on plant responses to this chitin tetramer, a low-molecular-weight chitin mix (CHL) enriched to 92% with dimers (2mer), trimers (3mer) and tetramers (4mer) was produced for potential use in biotechnological processes. Compared with untreated plants, CHL-treated plants had increased in vitro fresh weight (10%), radicle length (25%) and total carbon and nitrogen content (6% and 8%, respectively). Our data show that low-molecular-weight forms of chitin might play a role in nature as bio-stimulators of plant growth, and they are also a known direct source of carbon and nitrogen for soil biomass. The biochemical properties of the CHL mix might make it useful as a non-contaminating bio-stimulant of plant growth and a soil restorer for greenhouses and fields.

## 1. Introduction

Chitin is the second most abundant carbohydrate (after cellulose) in the biosphere. It is a nitrogen-containing polysaccharide that is generally composed of *N*-acetyl-d-glucosamine (GlcNAc, A) and d-glucosamine (GlcN, D) monomers bound by beta-1,4 linkages. Chitin is the major structural component of fungal cell walls and spores, crustacean shells, insect exoskeletons, mollusks and some protozoa. In the soil, chitin comes principally from insects and fungi. Millions of tons of chitin are discharged onto the sea floor annually as “marine snow” by copepod (planktonic crustaceans). The decomposition of chitin is very significant in the natural soil ecosystem, and it removes tons of chitin that accumulate every year from dead insects and later used by soil biomass [1]. The release of organically-bound nitrogen and carbon from chitin is an important factor that should be taken into account when investigating carbon and nitrogen cycling in ecosystems. Chitin is also the principal source of carbon and nitrogen for chitinolytic organisms, which are largely marine and soil bacteria belonging to the genera of the *Proteobacteria*, *Bacteroidetes*, *Actinobacteria* and *Firmicutes*, as well as soil fungi [2,3,4].

These chitin-decomposing organisms contain the metabolic machinery necessary to detect, modify and transport small chitin oligosaccharides (generally from two–four monomers long), and incorporate them directly into their glycolytic and nitrogen metabolic pathways as glucose (carbon) and ammonia, respectively [5,6,7].

Chitin is primarily converted by organisms to the more soluble biopolymer, chitosan, via a modification catalyzed by deacetylase enzymes that recognize a sequence of four GlcNAc units, one of which undergoes de-acetylation. Chitosan is not only a recognized antibacterial biopolymer [4], but it is also a source of nutrients for insects, bacteria and fungi living in the soil [8]. The biochemical properties of chitosan make it particularly useful for biomedical applications, such as wound dressing, weight loss agent, blood cholesterol control, surgical sutures, cataract surgery or periodontal disease treatment, and chitinolytic enzymes from bacteria and fungi are useful to pharmacological enterprises as a source of antifungal agents [9].

The exposure of plants to chitosan results in the activation of defense response genes associated with biotic stress [10], and chitosan is normally used in agriculture as an agent to induce innate plant protection [11]. In addition, the literature describes the use of chitosan to stimulate various plant growth parameters in potatoes (*Solanum tuberosum*), tomatoes (*Solanum lycopersicum*), orchids (*Orchidaceae*), grape vines (*Vitis vinifera*) or pines (*Pinaceae*) [11,12]. The stimulating effect of chitin or chitosan on plant growth has traditionally been attributed to the positive effects on soil biomass and on the association of symbiotic organisms with plants, more than to a direct effect on plant growth itself [4,13]. Additionally, high-molecular-weight chitin has been shown to produce an increase in eukaryotic and prokaryotic microflora when it is used in the soil as a source of nutrients [13,14,15,16].

In parallel, chitin, the principal component of fungal spores, is a well-characterized elicitor of plant responses, as it can activate the plant innate immune response by inducing the expression of genes related principally to a biotic stress in response to phytopathogenic fungi [17,18]. Plants can recognize high-molecular-weight chitin via specific receptors, which have been characterized in several plant species, including rice (*Oryza sativa*) [19], *Arabidopsis* (*Arabidopsis thaliana*) [20,21,22] and *Medicago* (*Medicago truncatula*) [23]. The chitin receptors implicated in plant defense, such as CERK1 and Lyk 5, can bind a chitin 8mer with higher affinity than smaller fragments [21,22,24,25]. Previously, we showed that a high-molecular-weight chitin mix (CHH) derived from crab shells and a purified chitin 8mer oligosaccharide induced a similar suite of genes (related to the defense response) in *Arabidopsis* seedlings [17,18]. Some of these genes were essential for a successful plant defense response against phytopathogenic fungi [26]. In soybean (*Glycine max*), compared with small oligomers of chitin, oligomers of chitin and chitosan that were larger than four monomers produced an increase in the production of phenolic compounds mediated by phenylalanine ammonia lyase [27].

Additionally, chitosan heptamers can target the chromatin within the plant nucleus, altering the chromatin conformation, which has been shown to change the expression and gene activation of the plant cell [28,29].

In contrast, short-chain oligomers of chitin (chitooligosaccharides, COs) have been found to be associated with non-stress-related plant responses. It was shown that while the foliar application of CHH decreased the net photosynthetic rate of maize (*Zea mays*) and soybean, this effect was not observed in plants treated with a chitin 5mer [30]. In addition, COs shorter than 6–7mers induced a lower production of reactive oxygen species in plants, compared to larger chitin oligomers [31]. Moreover, unlike CHH, COs were unable to induce the defense marker gene, mitogen-activated protein kinase 3 (*MAPK3*) [17] and to activate the defense-associated MAPK cascade [32,33]. More recently, it has been suggested that COs play an important role in the initiation of legume-*Rhizobium* symbiosis [34,35], and in the activation of the initial stages of root colonization by arbuscular mycorrhizal fungi [36]. Short forms of chitin oligosaccharides bound to lipids, which are known as lipochitooligosaccharides (LCOs), and nodulation (Nod) factors, were found to be secreted by rhizobial bacteria and mycorrhizal fungi during the establishment of symbiotic interactions, and they have been shown to increase the early plant growth of both soybean and maize [37,38].

In this study, we showed that treating plants with a chitin 4mer activated a transcriptional response in genes that were principally related to plant development and nitrogen and carbon metabolism. Our analysis revealed that this response differs significantly from previously-described plant defense-related response activated by a chitin 8mer or CHH [18] and by high-molecular-weight chitosan [10]. Additionally, we produced a CHL that was enriched with 2mer–4mers (92.3%) that produced a direct effect in vitro, activating plant growth and producing an increase in total nitrogen and carbon content compared with controls.

Our results point to a mechanism whereby naturally occurring low-molecular-weight forms of chitin might contribute not only to nutrient allocation in soil microorganisms, but also to the stimulation of plant development. These compounds could potentially be used as natural bio-stimulants of plant growth and soil restorers in agriculture.

## 2. Results

### 2.1. Analysis of Arabidopsis Transcriptional Response to the Chitin 4mer

In order to better understand the different types of plant responses induced by chitin in nature, we treated *Arabidopsis* seedlings with a highly purified chitin oligomer, specifically chito-fourmer (4mer), and we compared these results with our previous results associated with a chito-octamer (8mer) and a high-molecular-weight chitin mix (CHH) derived from crab shells [32]. The genomic response of the plants was then determined using Affymetrix full-genome microarrays (ATH1).

**Figure 1:**
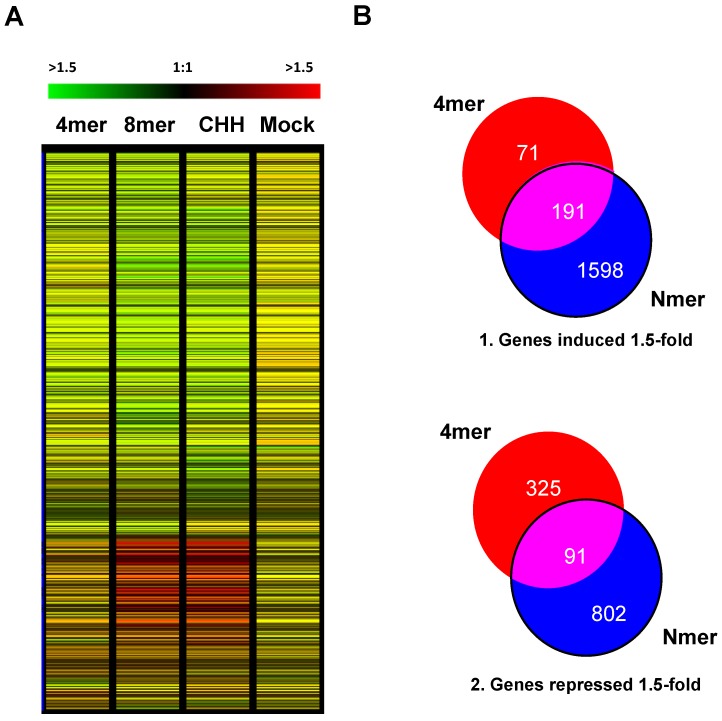
Microarray analysis of Arabidopsis after chitin treatments. (**A**) Hierarchical cluster of the ratio values of the genes that responded to different sizes of chitin, 4mer, 8mer and high-molecular-weight chitin mix (CHH). Each gene is represented by a single row, and each column represents an individual treatment. Red represents upregulated genes, green downregulated genes and black genes with no change (the signals are relative to the control treatment, which was water). (**B**) Venn diagrams of hierarchical clustering results; (**B1**) Venn diagram of genes showing a ≥1.5-fold increase in expression after 4mer treatment and 8mer or high-molecular-weight chitin (CHH) treatments (i.e., Nmer treatments); (**B2**) Venn diagram of genes showing a ≥1.5-fold decrease in expression after 4mer treatment and Nmer treatments. Two-way analysis of variance (ANOVA) was used for clustering. The genotypes and treatment groups were analyzed using a *p*-value of 0.5, with *p*-value > 0.5 = not significant and *p*-value < 0.5 = significant. Three array data replicates were used for the analysis.

Of the approximately 14,373 genes on the arrays, 5435 genes showed altered expression according to the Statistical Analysis of Microarrays program (SAM) (Figure 1A). In order to narrow down the list, genes that were induced or repressed by 1.5-fold were identified and grouped using Venn diagrams (Figure 1B). A set of 191 genes was induced in all three treatments (Figure 1(B1)), while a set of 91 genes was repressed in all three treatments (Figure 1(B2)). The chitin 4mer induced 71 genes specifically, and the CHH and 8mer treatments (Nmer) induced a much larger set of genes (1598). Interestingly, after treatment with the chitin 4mer, more genes that responded specifically to the 4mer treatment (rather than the Nmer treatments) were significantly repressed (325) than induced (71). Roughly equivalent numbers of genes were uniquely repressed by the chitin 4mer and CHH (339 and 329, respectively), while 239 genes were repressed by treatment with the chitin 8mer (and a total of 802 genes were repressed by the Nmer treatments; Figure 1(B2)).

In order to determine the enrichment of functional categories or overrepresented genes that responded to the chitin 4mer, genes induced greater than a 1.5-fold by the 4mer and not by other treatments were classified using three different in silico tools; the Bar Toronto Classification Super Viewer tool (http://bar.utoronto.ca/; Supplementary Materials Figure S1), the agriGO Tool [39], (http://bioinfo.cau.edu.cn/agriGO/) (Figure S2) and the PageMan tool from MapMan Software [40], (Figure S3). These analyses showed that genes and functional categories induced by the chitin 4mer belonged principally to the categories of developmental processes, cell organization, biogenesis, multicellular organismal development, membrane transport and primary amino acid metabolism (Figures S1 and S3). Among the genes repressed by the 4mer, genes related to biotic stress responses were overrepresented, and the proportion of these biotic stress-related genes (among all of the repressed genes) was higher than the proportion of induced biotic stress-related genes by the 4mer (Figure S3). These results are consistent with a non-biotic stress plant molecular response to the chitin 4mer, more closely related to the promotion of plant development and completely different from the previously-identified response to Nmer treatment, related to activation of plant defense and innate immunity [32].

### 2.2. Analysis of Chitin Mix Enriched with Low-Molecular-Weight Chitin Oligosaccharides 

**Figure 2:**
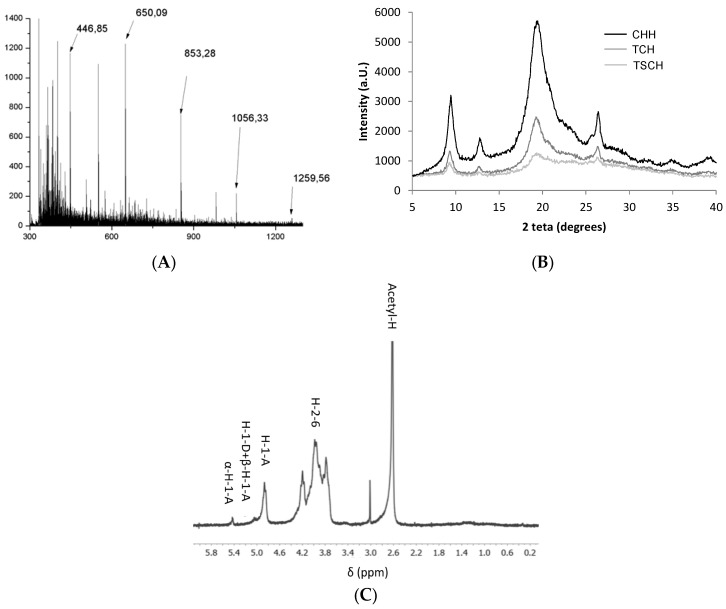
CHL sample characterization. (**A**) Matrix-assisted laser desorption/ionization time-of-flight (MALDI-TOF) Ultraflex profile of CHL mix in a 2,5-dihydroxybenzoic acid (DHB) matrix. The obtained molecular weights (OMW) are marked with arrows; (**B**) X-ray diffraction (XRD) pattern of the following chitin mixes: untreated chitin (CHH), thermally-treated chitin (TCH) and sonicated chitin after thermal treatment (TSCH). The intensity is in arbitrary units (a.u.), and 2θ degrees represents the diffraction angles; (**C**) Proton nuclear magnetic spectroscopy (1H-NMR) spectrum (300 MHz) of CHL in concentrated deuterium chloride (DCL) at room temperature. The signals of *N*-acetylglucosamine residues are marked.

Taking into account previous works [41,42,43,44,45,46], we produced a low-molecular-weight chitin mix (CHL) using combined thermal treatment and sonication on a high-molecular-weight chitin mix (CHH, see the Material and Methods Section). In order to characterize this CHL mix, several analyses were performed. First, to determine the composition of the CHL mix, the sample was analyzed using matrix-assisted laser desorption/ionization time-of-flight (MALDI-TOF) mass spectrometry. The mix of chitin fragments obtained by MALDI-TOF mass spectrometry is shown in Figure 2A. The MALDI-TOF spectrum of the chitin oligomers revealed that the sample was composed of chito-oligomers that were 100% acetylated with a degree of polymerization (DP) ranging from 2 to 6 (A2 to A6 at Table 1). The relative ion intensity of each signal can reflect the quantification of the products (Table 1, [47]). The theoretical molecular weight (TMW) and obtained molecular weight sizes (OMW) of the chitin fragments in the CHL mix and an estimation of the composition of the CHL mix are given in Table 1. The most abundant oligomers were A2, A3 and A4 (34.5%, 35.6% and 22.2%, respectively), then A5 and A6 at 6.48% and 1.20%, respectively, with no oligomers with a DP higher than six (Table 1). This 100% acetylated CHL mix was used for further experiments on plants.

Secondly, the effect of the thermal treatment and sonication on the samples was explored using X-ray crystallography (XRD). As seen in Figure 2B, both non-treated (CHH) and thermal-treated chitin (TCH) showed XRD patterns with strong reflections at 9.2° and 19.2° and minor reflections at 12.6°, 22.9°, and 26.2°. The results showed that the non-treated chitin (CHH) had a crystallinity index (CrI) of 73%, and this value was reduced to 67% after the thermal treatment as expected (thermally-treated chitin (TCH) in Figure 2B). Additionally, after the thermal treatment, plus sonication, (sonicated chitin after thermal treatment, TSCH sample in Figure 2B), the sample had a much lower CrI value (around 50%), and the intensity of the diffraction was less intense than that of CHH. Both the crystallinity pattern and CrI were in good agreement with those previously reported for α-chitin [48].

Finally, in order to confirm the presence or absence of larger molecular-weight chitin oligosaccharides in the CHL sample after the thermal treatment plus sonication, a proton magnetic spectroscopy (^1^H-NMR) analysis was performed. The ^1^H-NMR profile (Figure 2C) shows that the characteristic signals of acetyl protons and the ring protons were around 2.6 ppm and 3.6–4.5 ppm, respectively. The H-1 of internal acetylated units resonated at 4.91 ppm, while the characteristic resonances in the anomeric region of the acetylated α- and β-anomers were 5.43 and 5.05 ppm, respectively. Signals corresponding to *N*-acetylglucosamine residues at 5.07 (H-1), 5.65 (H-1 reducing end, α), 5.21 (H-1 reducing end, β), 3.44 (H-2), 3.57 (H-1 reducing end, α) and 3.32 (H-1 reducing end, β) were detected [49]. Based on this spectrum, a degree of polymerization (DP) of five was estimated.

These results confirmed that thermal treatment plus sonication induced the opening and breaking of the CHH biopolymer, forming the CHL mix enriched with A2, A3 and A4 fragments (a total of 92.3%, see Table 1), with no contamination with higher molecular-weight chitin oligosaccharides with more than six monomers.

### 2.3. Analysis of the Vegetative Growth of Chitin-Treated Arabidopsis 

As the microarray analysis of genes that responded to the chitin 4mer suggested that the 4mer mostly induced genes related to plant development and nitrogen and carbon metabolism, a study was designed to test whether small chitin fragments might produce a direct effect on plant growth. For these experiments, plants were treated with CHL under in vitro conditions, to control the composition of the growth medium (thereby avoiding the confounding factors that might occur in a greenhouse experiment). *Arabidopsis* seedlings were grown for 21 days under these in vitro conditions with a low concentration of nitrogen in the medium (see the Materials and Methods), in either the presence or absence of CHH [18,26] and CHL.

**Figure 3:**
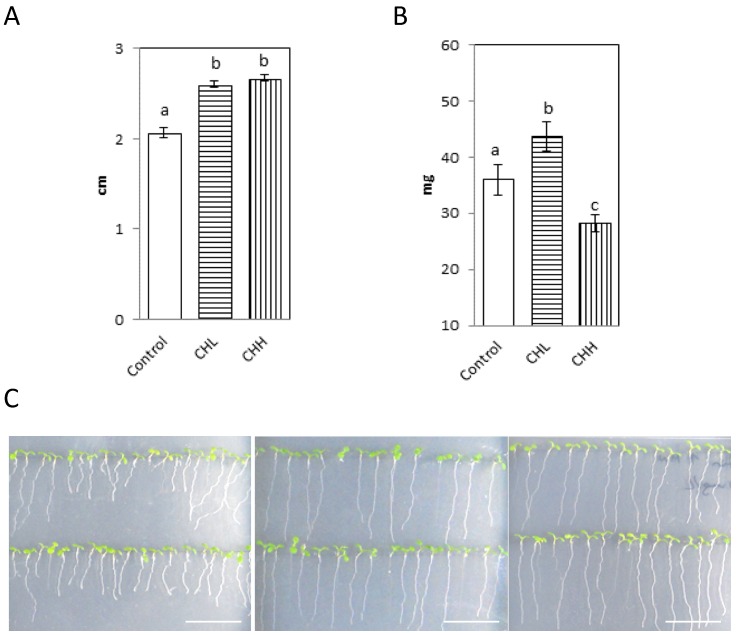
Chitin increased plant growth in vitro. (**A**) The radicle length of controls and plants treated with low-molecular-weight chitin mix (CHL) or high molecular weight chitin mix (CHH); (**B**) Fresh weight of controls and plants treated with CHL or CHH. The plants were grown for 20 days; (**C**) Representative plates of control seedlings (left), seedlings treated with CHL (center) and treated with CHH (right) after seven days. The experiments were performed at least three times with similar results. The data were analyzed using one-way analysis of variance (ANOVA) and the Statgraphics program Centurion XVI.II. Different letters indicate significant (*p*-value < 0.05) differences between treatment groups, according to Duncan’s test. Bars: 2 cm.

After 20 days, there was an increase in the radicle length of 6% in the CHL group treatment, and 11.5% in the CHH group treatment was observed in plants after 20 days, compared with the control (Figure 3A,C). The total plant fresh weight increased by 10% in the CHL group treatment compared with the controls, although the total plant fresh weight decreased by 10% in the CHH group (Figure 3B).

### 2.4. Analysis of the Total Content of Nitrogen and Carbon Content of Chitin-Treated Arabidopsis

**Figure 4:**
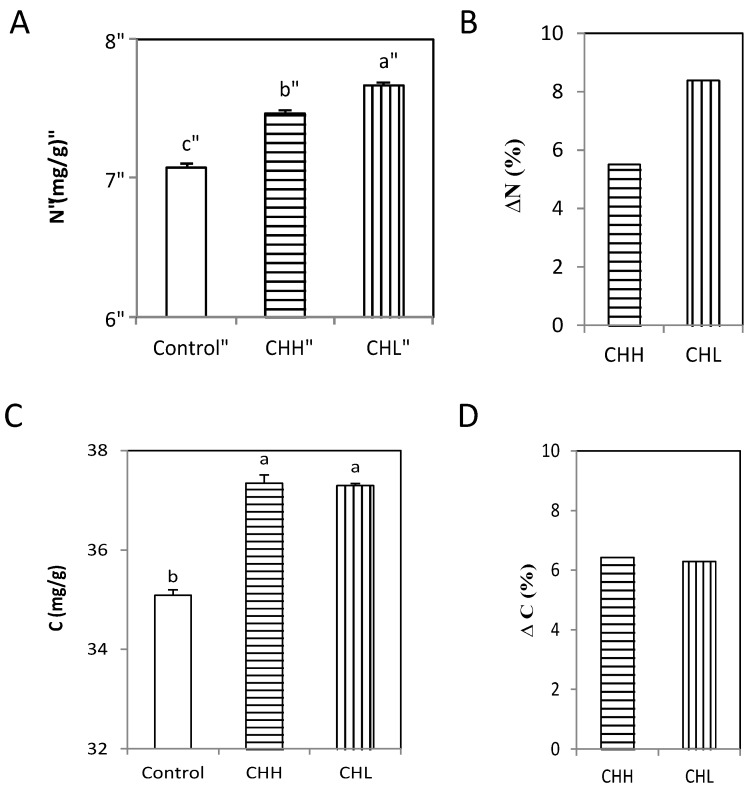
Chitin induces an increase of the total nitrogen and carbon content of *Arabidopsis* plants in vitro. (**A**) Total nitrogen content in controls (white bars) and plants treated with the low-molecular-weight chitin mix (CHL) or high-molecular-weight chitin mix (CHH); (**B**) Percentage of increase (ΔN) in total nitrogen content in plants treated with CHL and CHH relative to the controls; (**C**) Total carbon content in controls and plants treated with CHL and CHH; (**D**) Percentage increase (ΔC) in total carbon content in plants treated with CHL and CHH relative to the controls. Different letters indicate significant (*p*-value < 0.05) differences between treatment groups according to Duncan’s test. The measures were taken after 10 days of growth. The experiments were performed at least three times with similar results.

To determine whether chitin acted as a source of nutrients for the plants, total nitrogen and carbon were quantified in the CHL- and CHH-treated plants. An increase in plant total nitrogen content was observed in all treatments groups after 10 days, with increases of 5% in the CHH group and 8% in the CHL group compared to the controls (Figure 4A,B). A similar effect on total carbon content was observed in the CHL- and CHH-treated plants after 10 days, relative to the controls (Figure 4C), with no significant differences observed between the two treatment groups (Figure 4D).

### 2.5. Analysis of the Vegetative Growth of Chitin-Treated Poplar Explants

**Figure 5:**
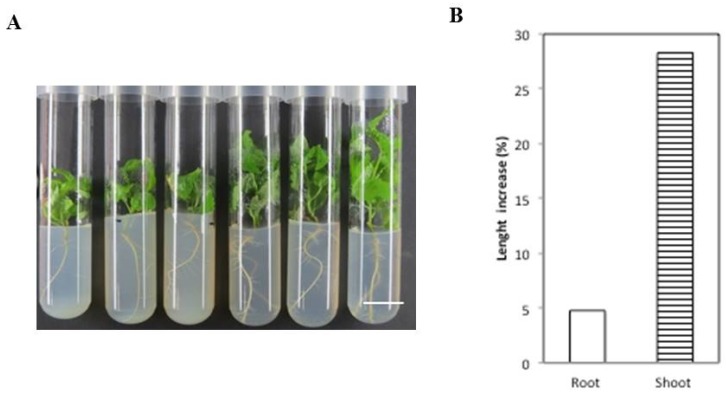
CHL produces an increase in shoot and radicle length in poplar explants. (**A**) Representative triplicates of poplar explants in the absence of CHL (in the three tubes on the left) or in the presence of CHL (in the three tubes of the right); (**B**) Increase in radicle and shoot length of explants grown in the presence of 100 µg/mL CHL compared to controls. The photos were taken after 45 days of growth. Bars: 2 cm.

In order to determine the effect of chitin on plant growth in other species, poplar explants of a *Populus trichocarpa* clone were grown under similar in vitro conditions for 45–70 days, following the same growth parameters as those in the *Arabidopsis* experiments (see the Materials and Methods). An increase in root length (up to 5%) and shoot length (up to 28%) was observed in the CHL-treated poplar explants relative to the controls (Figure 5A,B).

It was not possible, however, to experimentally determine the narrow range of exchange of the total nitrogen, carbon content and fresh weight of the treated poplar explants compared with the controls because of the high variability found between the plants in the measurements on these parameters. Additionally, because the poplar experiments allowed us to follow the plant growth for longer periods than the *Arabidopsis* experiments (as the *Arabidopsis* experiments always involved younger plants, before *Arabidopsis* transitioned to flowering), we explored longer-term poplar growth in the presence of CHL and CHH. However, leaf chlorosis and cell death were observed after 70 days (growth parameters for these experiments were not measured). These symptoms were not observed in the presence of CHL (Supplementary Figure S5).

## 3. Discussion

It has been extensively documented in the literature that high-molecular-weight chitin induces plant defense-related responses, protein phosphorylation and reactive oxygen species production at the molecular level [35]. In this study, we examined the expression profiles of *Arabidopsis* seedlings treated with a chitin 4mer and compared this response to plants treated with an 8mer and a high-molecular-weight chitin mix (CHH) using full-genome Affymetrix microarrays. Previously, we showed that treatment with a chitin 8mer elicited a host defense-related cellular response in *Arabidopsis* similar to that induced by CHH in *Arabidopsis* [18,26]. The induction of specific defense-related genes after treating *Arabidopsis* with CHH, has also been documented by other groups experimenting with *Arabidopsis* [21,50,51], rice and soybean [19,52,53,54]. Our results showed that chitin 4mer treatment induced an expression pattern that was distinct from the pattern observed after 8mer or CHH treatments. Interestingly, unique subsets of genes responded to each chitin treatment, suggesting that different chitin oligomer lengths activate different signaling pathways in *Arabidopsis*. While there was significant overlap in the expression patterns elicited by the 8mer and CHH, the gene expression patterns elicited by the 4mer were completely different. In silico analysis of the genes that were uniquely induced or repressed by the 4mer showed that the gene families that were overrepresented in the set of genes induced were not present in the set of repressed genes. These genes were related to lipid and nitrogen metabolism, nutrient physiology signaling and the transport of ammonium, sugars and potassium. In contrast, defense- and stress-related genes were overrepresented in the set of repressed genes, while a similar set of genes (i.e., stress response genes) was completely absent from the set of induced genes.

Analysis performed using the agriGO tool (http://bioinfo.cau.edu.cn/agriGO/) showed that the genes induced by the 4mer belonged principally to functional categories of developmental processes, cell organization, biogenesis, membrane transport and primary amino acid metabolism. A large number of genes related to biotic stress responses were overrepresented in the set of the genes that were repressed by the 4mer.

The 4mer induced several previously characterized development-related genes. These genes included *IPT5,* which has been implicated in stem cell initiation and meristem formation [55], *PTL* involved in auxin signaling [56], *EXPA22*, which has been implicated in cell elongation [57], *JAZ7*, which is involved in secondary growth [58], and ANAC101, which is involved in xylogenesis [59]. Other known genes that are known to be related to nutrient transport were also induced specifically by the 4mer, such as the sucrose transporter *AtSUC 7*, the Golgi sugar transporter *GONST5* [60,61], the *IRT1* iron transporter [62] and the amino acid transporter *AtPUP9* [63] (see selected genes in Table S1). In addition, several previously-characterized genes were associated with lower (but still upregulated) translational activation levels. These genes included genes related to xylogenesis [59,64], vascular patterning [65,66], early seedling development [67], cellular differentiation [68] and shoot development [69]. In parallel, genes that are known to be involved in inhibiting the activation of the jasmonic acid-mediated defense pathway in the absence of pathogens were also specifically induced by the 4mer, such as genes in the JAZ family [70].

However, there was no 4mer-induced upregulation of marker genes related to the defense response, such as pathogenesis-related protein 1, *PR1* (AT2G14610), defensin *PDF1.2* (AT5G44420), basic chitinase *PR3* (AT3G12500) and *MAPK3* (At3g45640). The transcriptional level of *MAPK3*, after 4mer treatment, was confirmed using qRT-PCR (see the Supplementary Materials). In addition, several unknown genes that belong to categories related to plant development were induced by the chitin 4mer. These changes in gene expression after 4mer treatment concur with the hypothesis that the exposure of a plant to low-molecular-weight forms of chitin might results in a molecular adaptation response and bio-stimulation of plant growth, rather than a stress response.

Small LCOs have recently been shown to produce an increase in root growth in maize [38]. We found that the transcriptional plant response to the chitin 4mer overlapped with the transcriptional response observed in maize in this previous study, especially regarding the over-represented gene families related to nutrient and ion transport, embryogenesis, secondary metabolism and gluconeogenesis. It should be highlighted that, according to the results of the study on maize, short-chain chitin oligomers and lipo-chitin oligomers may have overlapping roles in plant growth promotion and, perhaps, in plant-symbiont interactions. Other studies have shown that a chitin 4mer can serve as the backbone of Nod factors during the interaction between the symbiotic bacteria, *Rhizobia*, and the roots of legumes [34,35,71,72]. Additionally, short-chain chitin oligomers can trigger nuclear calcium spikes, a cellular event that also occurs also during Nod and mycorrhizal (Myc) factor-mediated symbiotic signaling [36]. The induction of symbiotic signaling by the arbuscular mycorrhizal fungal-produced LCOs and COs has been observed in both legumes and rice. In parallel, the detection of these LCOs by grasses and other non-legumes that act as hosts for arbuscular mycorrhizal fungi is potentially controlled by Myc receptors. Recently, it has been found that the intraradical colonization by arbuscular mycorrhizal fungi triggers the induction of a new LysM-type LCO receptor, LYS11 [73]. In addition, in *Medicago truncatula*, a high-affinity LCO-binding protein (LYR3) interacted with a key symbiotic receptor (LYK3) [74]. The detection of LCO during the establishments of legume-Rhizobium symbiosis is controlled by a LysM receptor-like kinase known as nodulation factor perception (NFP) in *M. truncatula* [75]. In rice, OsCERK1 was found to regulate both chitin-triggered immunity and symbiosis with arbuscular mycorrhizal fungi [76]. LCOs can modulate plant host immunity to enable endosymbiosis [77], and the ability to detect LCOs might have evolved from plant innate immunity signaling [35].

Additionally, chitosan oligomers can target the chromatin within the plant nucleus, altering the chromatin conformation and gene expression of the plant cell [28,29]. Although chitosan has a high affinity for DNA and chitin does not, a still unknown effect of chitin oligomers in the plant cell should not be discarded. All of these data indicate that the role of small COs in nature related to plant growth still needs more biochemical clarification.

The results from the in vitro experiments of plants grown in the presence of CHL and CHH might be in line with the hypothesis that these oligosaccharides might have a bio-stimulating effect on plant growth. This hypothesis is further supported by the transcriptional data, since the genes that responded differentially were related to ammonium, amino acid and glutamate metabolism, the hexosamine biosynthetic pathway and nutrient transport. However, additional experiments are necessary to determine the validity of this hypothesis. It is still unclear whether the observed effects on development are only a consequence of the observed transcriptional activation.

A previous study indicated that a highly purified chitin 5mer was able to increase root length in *Arabidopsis*, although the mechanism behind this effect remains unknown [78]. The increase in root length produced by treatment with CHL in this study was similar to that observed in the study on the chitin 5mer (i.e., around a 25% increase compared with controls). The 5mer comprised only 6.48% of the CHL, and there was a higher percentage (a total of 92.37%) of 2mer (dimer), 3mer (trimer) and 4mers (tetramer), so it is plausible that the principal effect observed in both studies was a consequence of a response specifically to fragments of five or fewer monomers.

We also observed that poplar explants were able to grow in medium containing only CHH as a source of carbon, although leaf chlorosis and cell death were observed after long periods of growth under in vitro conditions. Interestingly, these symptoms were not observed in the presence of CHL. The stress-response symptoms were also not observed in in vitro experiments involving *Arabidopsis* grown in the presence of CHH (but not in the presence of CHL). This may be because our experiments and analysis in *Arabidopsis* experiments were always performed using younger plants (in comparison with the poplar assays) well before *Arabidopsis* transitioned to flowering.

It is reasonable to hypothesize that plants may be able to degrade CHH, thereby releasing smaller fragments of chitin that could promote plant development (which may have happened in the case of treatments with CHL) or be used as a nutrient source for both the plant itself and the soil microorganisms present in the rhizosphere. This process would require a prior stress-response activation to induce chitinases production, thereby putatively leading to a negative trade-off concerning plant development. However, more detailed experiments are needed to determine whether this is the case.

It should be noted that previous studies have shown that plants can respond to CHH with increased expressions of innate immunity- and defense-related genes with induction ratios of more than 200-fold compared with controls [18,26]. However, the plant transcriptional changes produced by the chitin 4mer in the present study were much lower, with no inductions above eight-fold compared with the controls, and the genes induced by the 4mer were related principally to plant development, with a suppression of the genes involved in biotic stress. These results are in line with a molecular mechanism of adaptation response (rather than a transcriptional response to stress in order to aid survival), and the results suggest that the CHL described in this study might be useful as a bio-fertilizer, or, at a minimum, a bio-stimulator of plant development when combined with other nitrogen, phosphate and magnesium (NPK) fertilizers currently used in agriculture.

It is also important to determine whether plants possess the molecular machinery needed to transport COs and use them as a direct source of carbon and nitrogen, similar to chitinolytic microorganisms [5]. It is obvious that the energetic cost associated with the production of chitinases and for the subsequent transport and assimilation of chitin fragments by a plant would be higher than the energetic cost required to transport and assimilate small oligosaccharides. This fitness cost might explain why the increase in total nitrogen content observed in the CHL treatment-plants was higher than in the CHH-treated plants. Analogously, based on the microarray data, the stress response induced by the chitin 8mer and CHH may be associated with the cost of the synthesis of chitinases, which was not observed after the 4mer treatment. This fact might explain the stress that CHH treatment produced on poplar explants after longer periods of growth, which was not observed in CHL-treated plants. Our experiments showed that several chitinases were highly induced by the 8mer and CHH, but not at all by the 4mer. However, at the time points tested, no significant difference was observed in total carbon content or radicle length between the 4mer and 8mer/CHH treatments, although there were increases in comparison to the controls. The increase in growth seen in the CHH group might be attributable to the degradation of CHH oligosaccharides by the plant, and the energetic costs associated with this process might make CHH a less efficient bio-stimulator compared with CHL.

Although the molecular mechanism behind increased plant growth in the presence of CHL is still unknown, plants exhibit well-known chitinolytic activity in response to phytopathogens [79], sharing similarities with chitinoclastic organisms. Chitinoclastic activity has been described previously in several microorganisms, including *Vibrio furnissii* [5], *Vibrio carchariae* [80], *Amycolatopsis orientalis* or *Kitasatospora* sp. [81], *Serratia marcescens* [82], *Escherichia coli* [83] and *Streptomyces coelicolor* [84]. The chitinoclastic cascade in *Vibrio* has been characterized [6] and found to involve 10 genes that are implicated in chitin catabolism. *Vibrio* is able to incorporate the chitin-derived glucose into the glycolytic pathway and the amino groups into amino acids [5,85]. The presence of orthologous genes related to a chitinolytic pathway in plants and the induction by the chitin 4mer, but not by 8mer or CHH of *Arabidopsis* genes related to amino acid, sugar and ammonium transport (Figure S1), indicate that plants might utilize the amino groups derived from chitin as a source of nitrogen. However, this response might only involve transcriptional stimulation of plant growth rather than the stimulation of a chitinoclastic pathway. Along the same lines, the 4mer slightly induced enzymes that are related to glutamine and glutamate synthesis, a mechanism that is known for its roles in the induction of cell growth and as a nutrient-responsive signaling pathway [86].

As commercially available purified small chitin 4mers and chitosan fragments or mixes are extremely expensive to be used in the high volumes, in this work, we developed a less expensive method to obtain a chitin mix, enriched with low-molecular-weight oligosaccharides. This method could be used in the future in greenhouses and fields. The mainstream process for obtaining chitin- or chitosan-derived oligomers involves acid hydrolysis, several deproteinization steps (depending on the natural origin of the chitin), deacetylation and purification by high-performance liquid chromatography, which can lead to the production of oligomers with very high levels of purity. In addition, if chitin oligomers are required, chitosan oligomers need to be re-acetylated [41,42]. As we were interested in producing a mixture of chitin fragments with as high a degree of acetylation as possible and a low molecular weight, we optimized a protocol based on previous work that indicated that chitin biopolymers can be fragmented using sonication. Previous studies indicated that sonicated chitin is more susceptible to chitinase activity than the non-treated biopolymer [39,40]. Sonication of chitosan also decreased its molecular weight and crystallinity grade [43]. In addition, we observed that other studies showed that heating chitin during the depolymerization process made it more accessible to the action of chitinase [44]. Both processes had no effect on the functional groups found in the polymers. Our newly-developed protocol is a quick method that allows CHL to be produced at a low cost, which means that CHL could potentially be used in agriculture and industry, something that might be impossible with previous biochemical methods used to obtain small forms of chitin oligosaccharides.

Our present and previous results highlight the high variability of chitin-derived compounds in nature, making the study of plant responses to chitin very complex. Additional experiments are currently in progress. These experiments aim to determine the activity of CHL on soil, greenhouse and field conditions and the molecular mechanisms that allow plants to respond differentially to different chitin oligosaccharides in natural conditions, in order to determine the biotechnological potential of the CHL mix for promoting plant growth in the field and to be used as a bio-stimulator of plant development.

## 4. Materials and Methods

### 4.1. Plant Growth and Chitin Treatments

For the microarray analysis, *Arabidopsis thaliana* (*Col-0* ecotype, obtained from Arabidopsis Biological Resource Center stock) seeds were treated according to a previously-reported procedure [26], with small modifications. The seeds were surface sterilized and grown in liquid Murashige and Skoog culture medium at a density of approximately 500 seeds (10 mg) per 125-mL flask. The flasks with the seeds were incubated at 4 °C for 6 days and then placed in a shaking incubator at 150 rpm for 2 weeks under constant illumination (125 µmol·m^−2^·s^−1^) at 23 °C. After 14 days, the seedlings were treated with purified chitin oligo-4mer (4mer, Seikagaku Corporation, Tokyo, Japan) at a final concentration of 100 µg/mL. After 30 min of treatment, the seedlings were harvested, flash-frozen in liquid N_2_ and stored at −80 °C until analysis.

For the in vitro *Arabidopsis* experiments, *Arabidopsis thaliana* (*Col-0* ecotype, obtained from ABRC stock) seedlings were surface sterilized (30% bleach and 0.01%, sodium dodecyl sulfate for 20 min), stratified (i.e., cold treated) for 2 days at 4 °C and placed in small square petri dishes containing 70 mL of 1/2× Murashige and Skoog (MS) Basal Salt Mixture (2.28 g/L, modification 1B micro and 1/2 macro elements including vitamins, # M0233.0050, Duchefa Biochemie, Haarleem, The Netherlands) plus 2% sucrose at pH 5.8. The plates were then transferred into a growth chamber. The medium contained half of the nitrogen present in standard media (825 mg/L, 10.3 mM).

For in vitro poplar experiments, the hybrid poplar *Populus tremula × P. alba INRA clone 717 1B4* was used; explants of 3–4 cm obtained from 60-day-old plants were transferred into glass tubes with 15 mL of the MS medium described above and placed into an in vitro chamber. The *Arabidopsis* seedlings were grown for 21 days and poplar explants for 70 days at 60% humidity (*v*/*v*), temperatures of 24 °C during the day and 22 °C during the night, with a 14-h light/10-h dark photoperiod and a light intensity of 150 µE·m^−2^ per s for all experiments.

In order to obtain the CHL mix for the CHL treatments, ultrapure chitin from shrimp shells (Cat#C9752, acetylation degree higher than 95% Sigma-Aldrich, St. Louis, MO, USA) was finely ground using a grinder and a porcelain mortar to obtain a homogeneous mixture of chitin. This was then suspended in ddH_2_O (1–4 g/L, Milli-Q purity grade) and autoclaved for 20 min at 121 °C. Once the solution was cooled to room temperature, the mix was subjected to sonication (50 Hz) for 15 min at a temperature lower than 25 °C. The suspension was then stored at 4 °C until further use or lyophilized for further analysis.

### 4.2. Oligomer Characterization

For CHL characterization, a known amount of the filtrate of the CHL solution was initially analyzed, and no soluble oligomers were detected in the liquid. The sample was then freezing dried.

XRD patterns were obtained using a Bruker D8 Advance diffractometer with CuKa radiation (step size, 0.05, counting time, 3.5 s). Sample crystallinity (CI) was determined using the following equation previously described [87]: *CI* (%) = [*(I*_110_ − *I_am_)*/*I*_110_] × 100, where *I*_110_ (arbitrary units) is the maximum intensity of the (110) peak at around 2θ = 19° and *I_am_* (arbitrary units) is the amorphous diffraction at 2θ = 12.6°.

^1^H-NMR spectra were recorded using a Varian spectrometer at 300 MHz (spectral width = 8000 Hz, number of transients = 128, block size = 4, recycle delay = 5 s). The mean polymerization degree (DP_n_) and the fraction of acetylated units (FA) were calculated using a previously described method by [47].

For the ^1^H-NMR measurements, a sample was dissolved at 4 °C in concentrated deuterium chloride (Sigma-Aldrich) at a concentration of 15 mg/mL. DPn was calculated based on the integrated area associated with all of the H1 protons divided by the integrated area associated with the reducing end protons. The mass spectra were recorded using a Bruker Ultraflex (Bruker Daltonik, Bremen, Germany) with MALDI-TOF/TOF equipment in positive-ion mode. For ionization, 2,5-dihydroxybenzoic acid was used as the matrix. The oligomers were soaked in a mixture of 1:1 water:methanol and mixed with the matrix prior to the analysis.

### 4.3. Microscopy and Photography Techniques

A stereomicroscope (MZ9, Leica Microsystems, Leica, Deerfield, IL, USA) with a charge-coupled device (CCD) camera (DC 280, Leica Microsystems) was used to obtain photos of the *Arabidopsis* seedlings growing on the plates. Image processing was performed using the ImageJ Software [88].

### 4.4. Nitrogen and Carbon Content Analysis

For each plate, 12 seedlings per treatment group were collected and grouped as a single sample. The aerial parts of each plant were separated from the roots, cleaned and dried in an oven at 65–70 °C for at least 48 h. The dried tissues were finely ground (to sizes less than 150 µm) in a porcelain mortar. The concentrations of nitrogen and carbon were determined using a mass elemental analyzer for macro-samples (LECO CHN-600, Leco Corp. St. Joseph, MI, USA) according to the manufacturer’s instructions.

### 4.5. Data Analysis of Growth Parameters

All statistical analyses were performed using StatGraphics Centurion XVI.II (StatPoint Technologies, Inc., Warrenton, VA, USA). A one-way analysis of variance (ANOVA) and Duncan’s mean comparison test were performed for the all experiments or *t*-test with a significance level of 0.05%. In the case of non-homogeneous variance, a nonparametric Kruskal–Wallis test was applied.

### 4.6. Microarray Preparation, Hybridization and Data Extraction

Total RNA samples were processed according to the manufacturer’s protocols with the following modifications (Affymetrix GeneChip Expression Analysis Technical Manual, Affymetrix, Inc., Santa Clara, CA, USA). Single-stranded, then double-stranded cDNA was synthesized from the polyA^+^ mRNA present in the isolated total RNA (20 µg of total RNA was used as the starting material in each sample reaction) using the SuperScript Double-Stranded cDNA Synthesis Kit (Invitrogen Corp., Carlsbad, CA, USA) and custom poly (T)-nucleotide primers that contained a sequence recognized by T_7_ RNA polymerase. The resulting double-stranded cDNA was used as a template to generate biotin-tagged cRNA from an in vitro transcription reaction, using the Bio-Array High-Yield RNA Transcript Labeling Kit (Enzo Diagnostics, Inc., Farmingdale, NY, USA). In accordance with the prescribed protocols, 20 µg of the resulting biotin-tagged cRNA were fragmented to strands of less than 100 bases in length following prescribed protocols (Affymetrix GeneChip Expression Analysis Technical Manual, Affymetrix, Inc., Santa Clara, CA, USA). The 20 µg fragmented target cRNA (20 µg) were hybridized at 45 °C with rotation speed of 60 rpm for 16 h (Affymetrix GeneChip Hybridization Oven 640) with the probe sets present on an Affymetrix ATH1 GeneChip array. The GeneChip arrays were washed and then stained (with streptavidin-phycoerythrin) on an Affymetrix Fluidics Station 400, followed by scanning on a Hewlett-Packard GeneArray scanner (Hewlett-Packard, Palo Alto, CA, USA). Three independent biological replicates were performed for each sample. Image analysis and pixel intensity were quantified using MicroArray Suite 5.0 software (Affymetrix, Inc., Santa Clara, CA, USA). Text files were then generated and exported to TM4 Microarray Software swift Version MeV 4.9 (http://www.tm4.org/index.html) for normalization and further analysis.

The data discussed in this publication have been deposited in the U.S. National Center for Biotechnology Information’s Gene Expression Omnibus (GEO) database [89] (see online resources below).

### 4.7. Microarray Data Analysis

The data were filtered and analyzed using the Statistical Analysis of Microarrays (SAM) program [90]. Genes identified as significant using SAM were exported and clustered using GeneSpring 6.0 (Figure 2A). Text files containing raw data were imported to TMEV [91] and were normalized as follows. First, values below 0.01 were set to 0.01, and then, each measurement was divided by the measurement at the 50th percentile of all of the measurements in the sample. The specific samples were then normalized to one another: three replicates of each treatment were normalized against the median of the control samples (water treatment). Each measurement for each gene in the specific samples was divided by the median of that gene’s measurements in the corresponding control samples. The raw data on all of the genes were then extracted and analyzed for significance using the Statistical Analysis of Microarrays (SAM) software program [90]. Genes determined to be statistically significant were listed, and the resulting information was imported into TMEV for further analysis. The genes were grouped by their biological function according to their gene ontology (GO) annotation using the Bar Toronto Super Viewer tool (Figure S1), MapMan tool (Figure S2; [40]) and agriGO toolkit (Figure S3; [39]).

### 4.8. Quantitative Reverse Transcription-PCR Analysis for Microarray Data Validation

Total RNA was isolated from the frozen plant tissues using TRIzol Reagent (Invitrogen^®^, Carlsbad, CA, USA) according to the manufacturer’s protocol. The RNA samples were treated with RQ1 DNase (Promega, Madison, WI, USA). Trace amounts of genomic DNA were removed by digestion with Turbo DNA-*free*™ (Ambion, Austin, TX, USA). First-strand cDNA synthesis was primed using an oligo (dT)_15_ anchor primer, and cDNA was synthesized using a First-Strand Synthesis Kit (Amersham-Pharmacia, Rainham, UK) according to the manufacturer’s protocol. An aliquot of 1.5 µL of the first-strand synthesis reaction was used as the template for PCR amplification. To ensure that the sequence amplified was specific, a nested PCR was performed using 1 µL of a 1:50 dilution of the products synthesized in the first PCR reaction as a template. The RT-PCR, PCR and nested PCR program consisted of: 3 min at 96 °C, 40 cycles of 30 s at 94 °C, 30 s at 65 °C and 1 min at 72 °C. The final extension step consisted of 7 min at 72 °C. The amplified PCR fragments were visualized using 1.5% agarose gels.

The qRT-PCR experiments were performed using a SYBR^®^ Green qPCR kit (Finnzymes, Espoo, Finland) with reactions at a final volume of 20 µL per well and using the cycle protocol recommended by the manufacturer. The samples were run in a DNA Engine Opticon^®^ 2 System instrument with the PTC-200 DNA Engine Cycler and CFD-3220 Opticon™ 2 Detector (Bio-Rad, Hercules, CA, USA). Gene-specific primers were designed using the Primer Express 2.0 program (Applied Biosystems, Foster City, CA, USA), and minimal self-hybridization and dimer formation of primers were determined using the Oligo 6.0 program (Molecular Biology Insights, West Cascade, CO, USA). Primers with annealing temperatures of 62 °C–65 °C that amplified products with lengths of about 300 bp were selected and then verified for specificity using Basic Local Alignment Search Tool (BLAST) searches. The efficiency of amplification for each gene was calculated as recommended by the manufacturer (Bio-Rad, Hercules, CA, USA). The following gene specific primers were used for quantitative RT-PCR: β*-ACTIN* (At3g18780): 5′-GTGATGAAGCACAATCCAAG-3′ (forward) and 5′-GAACAAGACTTCTGGGCAT-3′ (reverse); *MAPK3* (At3g45640): 5′-ATGAACACCGGCGGTGGCC-3′ (forward) and 5′-GGCATTCACGGGGCTGCTG-3′ (reverse); *ATSUC9* (At5g06170) 5′-AGCCGTTGGTTTCTTCGT-3′ (forward) and 5′-CTAATCACTCCAATAACAAG-3′ (reverse); *ATJAZ7* (At2g34600) 5′-CGGATCCTCCAACAATCC-3′ (forward) and 5′-GACAATTGGATTATTATG-3′ (reverse); *ATECP31* (At3g22500) 5′-GTCGAAGCACCTGATGTAGC-3′ (forward) and 5-GAGCAATGACGTTGGTACC-3′ (reverse); *ATEXPA22* (At5g39270) 5′-GTCGAAGCACCTGATGTAGC-3′ (forward) and 5′-CCACAAGCTCCCTGTTGAG-3′ (reverse). Data acquisition was performed using the Opticon Monitor Analysis software (Version 2.01), and changes in the transcript levels were determined using the 2^−ΔΔCT^ method [92]. Data points were compared using *t*-tests. Three independent biological replicates were used in each experiment.

### 4.9. Bioinformatics Analysis

Additional information about gene expression and data analysis tools was obtained from the following webpages: http://affymetrix.arabidopsis.info/narrays/, https://www.genevestigator.ethz.ch/, http://aramemnon.botanik.uni-koeln.de/, http://www.us.expasy.com/tools/, http://www.ncbi.nlm. nih.gov/ and http://www.ebi.ac.uk/Tools.

## 5. Conclusions

Using genomic approaches, we have identified several plant genes that respond to low-molecular-weight chitin-derived oligosaccharides. We identified a genomic and physiological link between transcriptional activation and plant development and a concomitant increase in plant growth and nutrient content in vitro. In addition, this study demonstrates the power of microarray data to identify potential transcriptionally activated metabolic networks in order to characterize novel signaling pathways. Our results concur with those of previous studies that show that, in nature, plants might activate a developmental response if small chitin fragments are present in the rhizosphere. Additional work is in progress to determine the exact molecular pathways that allow these compounds to stimulate plant development and their role in the natural environment.

## Figures and Tables

**Table 1:** Estimated composition of CHL mix. A comparison between Theoretical Molecular Weights MNa^+^ (TMW) and Obtained Molecular Weights (OMW) is shown. The corresponding intensity and percentage of each oligosaccharide in the mix CHL obtained by MALDI-TOF shown at Figure 2A is also indicated, Amer: number of *N*-acetylglucosamine oligosaccharides.

Amer	TMW (*m*/*z*)	OMW (*m*/*z*)	Intensity	%
A2	446.85	447.16	1166	34.53
A3	650.09	650.24	1203	35.63
A4	853.28	853.31	750	22.21
A5	1056.33	1056.39	219	6.48
A6	1259.56	1259.47	38	1.12

## Supplementary Materials

The following are available online at www.mdpi.com/1660-3397/15/2/40/s1. Figure S1: In silico data analysis of genes induced by the 4mer, Figure S2: Functional classification of genes specifically induced by the 4mer, Figure S3: Overrepresentation analysis of genes differentially induced or repressed by the 4mer, Figure S4: Verification of microarray results for selected genes responding differentially to 4mer measured by qRT-PCR, Figure S5: Poplar growth under different conditions into the medium, Table S1: Data represent the corresponding array signal values on selected genes analyzed by qRT-PCR (Figure S4), Table S2: Selected known development related genes specifically induced by the 4mer more than 1.5 related to controls. Online Resources: U.S. National Center for Biotechnology Information’s Gene Expression Omnibus (GEO) database. Accession Number: GSE83858 (https://www.ncbi.nlm.nih.gov/geo/query/acc.cgi?acc=GSE83858).
