# Supplementary material for: Short-Chain Chitin Oligomers: Promoters of Plant Growth
Source: Mar Drugs. 2017 Feb 15;15(2):40. doi: 10.3390/md15020040 (PMC5334620; doi:10.3390/md15020040)
Supplement: Supplementary file 1 [file marinedrugs-15-00040-s001.pdf]

Supplementary Materials: Short-chain Chitin Oligomers: Promoters of Plant Growth

Alexander J. Winkler, Jose Alfonso Dominguez-Nuñez, Inmaculada Aranaz, César Poza-Carrión, Katrina Ramonell, Shauna Somerville and Marta Berrocal-Lobo

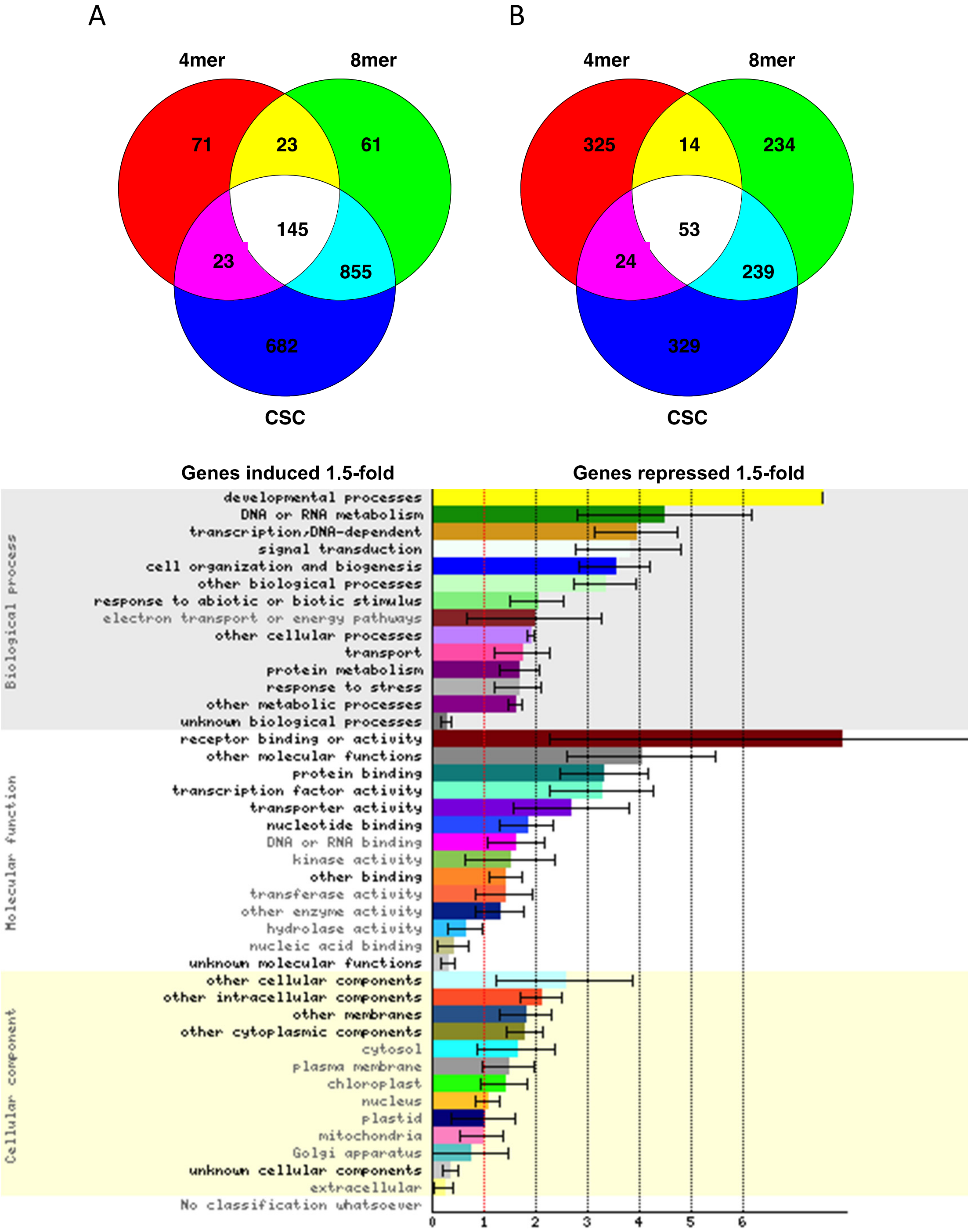

C

Figure S1. *In silico* data analysis of genes induced by the 4mer. (**A**) Venn diagrams distribution of subsets of overlapping genes after 4mer, 8mer or CHH treatment, showing ≥1.5-fold change in expression; (**B**) Venn diagrams distribution of subsets of overlapping genes showing ≤1.5-fold change in expression after 4mer, 8mer or CHH treatment. The statistic used for clustering wastwo-way

ANOVA. Genotype and treatment groups were analyzed using a *p*-value of 0.5 with $p > 0.5$ considered not significant; $p < 0.5$ considered significant. Three array sample replicates were used for theanalysis. (**C**) Classification Superviewer of genes specifically induced by the 4mer. Data analysis was performed using Bar Toronto Gene Onthology tool (http://bar.utoronto.ca/).

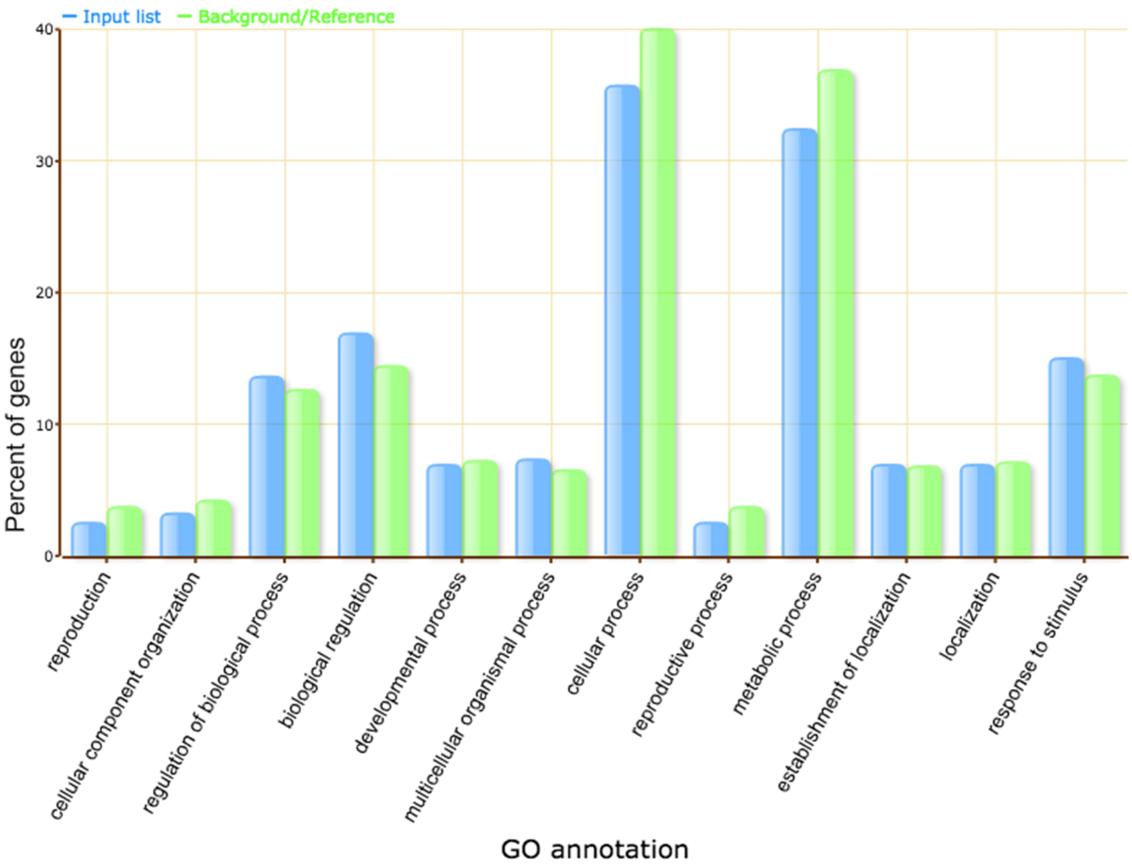

Figure S2. Functional classification of genes specifically induced by the 4mer. Data analysis using Agrigo Tool. (http://bioinfo.cau.edu.cn/agriGO/).

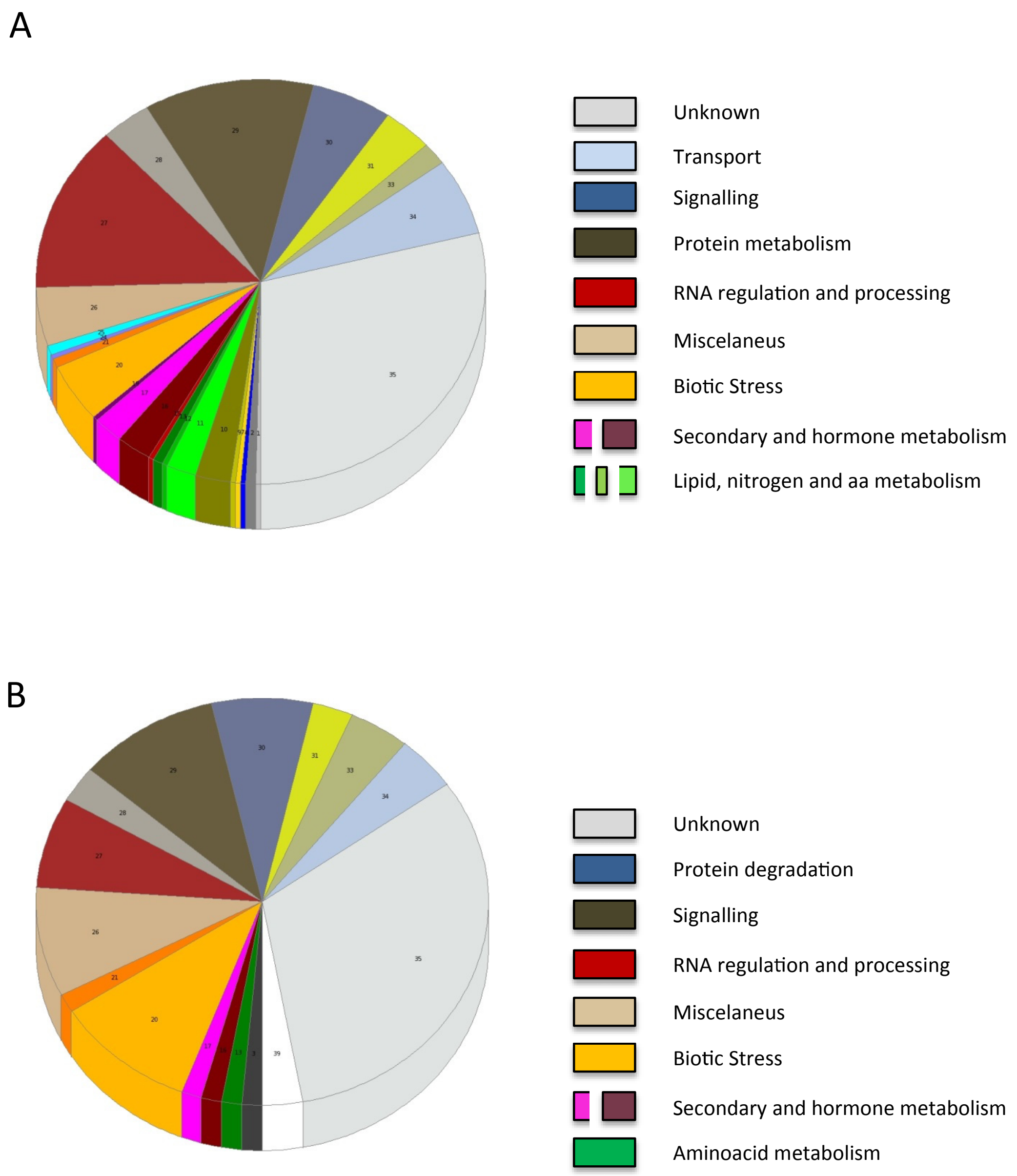

Figure S3. Overrepresentation analysis of genes differentially induced or repressed by the 4mer. Over-represented functional categories are colored. A. Categories of genes differentially induced by 4mer (numbered from 1 to 35). B. Categories of genes differentially repressed by 4mer (numbered from 1 to 39). The analysis was performed within functional categories using PageManTool of MapMan Software [1].

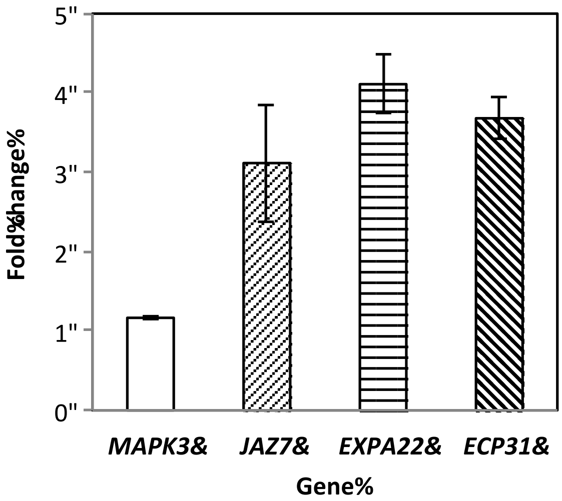

Figure S4. Verification of microarray results for selected genes responding differentially to 4mer measured by qRT-PCR. Col-0 wild-type plants were analyzed after 30 minutes of chitin 4mer treatment. Genes with different patterns of expression between treatments were selected for confirmation purposes. Fold change on selected genes: *MAPK3* (At3g45640), *SUC9* (At5g06170), *JAZ7* (At2g34600), *EXPA22* (At5g39270), *ECP31* (At3g22500) and β-*Actin2* (At3g18780). Data represent the fold signal related to mock plants (-2DDCt. [2]).

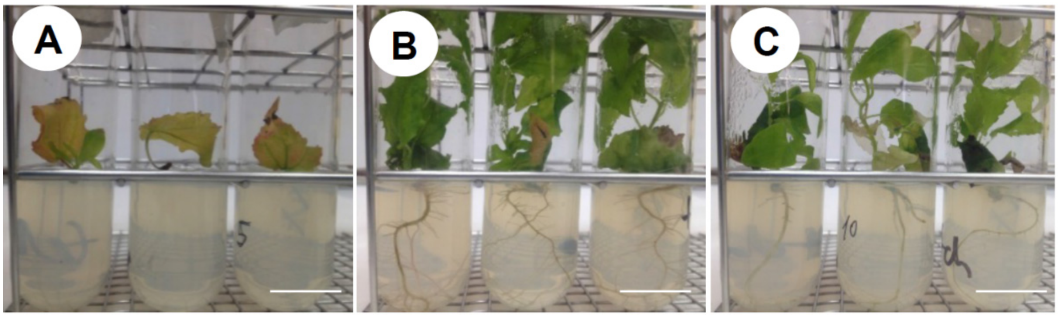

Figure S5. Poplar growth under different conditions into the medium. (**A**) Medium in absence of sucrose or CHH, (**B**) medium in presence of 2% Sucrose, (**C**) medium in presence of 100 mg/L CHH. Photos were taken after 70 days. Leave chlorosis or cell death were not observed after 40 days of growth in any plant (data not shown). Bars: 2 cm.

Table S1. Data represent the corresponding array signal values on selected genes analyzed by qRT-PCR (Figure S4). Averaged data from the three replicates of the microarray experiment for these genes and the SD are also shown.

Gene name	ID	Array Signal
MAPK3	At3g45640	1.04 ± 0.7
JAZ7	At2g34600	2 ± 0.6
EXPA22	At5g39270	1.6 ± 0.4
ECP31	At3g22500	2.1 ± 0.2

Table S2. Selected known development related genes specifically induced by the 4mer more than 1.5 related to controls. Reference publication and Gene Ontology functional classification (GO) of each gene is also presented (http://geneontology.org/).

Locus ID/ Probe	Annotation	Reference and/GO classification
At5g19040/249972_at	*IPT5*	Cheng et al., 2013/ meristem formation
At5g03680/250908_at	*PTL*	Lampugnani et al., 2013/ Aux signaling
At1g66570/256407_at	*SUC7*	Sauer et al., 2004/ carbohydrate transport
At1g02790/262122_at	*PGA4*	Jiang et al., 2013/ pollen development
AT5G40420/249353_at	*OLEO2*	Deruyffelaere et al., 2015/ olibody biogenesis
At5g39270/249501_at	*EXPA22*	Irshad et al., 2008/ cell elongation
At3g25905/258082_at	*CLE27*	Czyzewicz et al., 2015/ root arquitecture
At4g19690/254550_at	*IRT1*	Boonyaves et al., 2015/ iron transporter
At1g21870/260850_at	*GONST5*	Handford et al., 2004/ golgi sugar transporter
At1g18220/256124_at	*PUP9*	Gillisen et al., 2000/ aminoacid transporter
At2g34600/266901_at	*JAZ7*	Sehr et al., 2010/ growth, JA pathway inhibition
At3g51895/246310_at	*SULTR3:1*	Cao et al., 2013/ sulfate transporter

References

1. Usadel, B.; Nagel, A.; Steinhauser, D.; Gibon, Y.; Blasing, O.E.; Redestig, H.; Sreenivasulu, N.; Krall, L.; Hannah, M.A.; Poree, F.; et al. PageMan: An interactive ontology tool to generate, display, and annotate overview graphs for profiling experiments. *BMC Bioinform.* 2006, *7*, 535.
2. Livak, K.J.; Schmittgen, T.D. Analysis of relative gene expression data using real-time quantitative PCR and the 2(T)(-Delta Delta C) method. *Methods* **2001**, *25*, 402–408.
